# Fear of Sleep in the Acute Aftermath of Trauma Predicts Future Posttraumatic Stress Disorder: The Moderating Role of Community Violence Exposure

**DOI:** 10.3390/bs16030443

**Published:** 2026-03-18

**Authors:** Anthony N. Reffi, Tanja Jovanovic, David A. Kalmbach, Kristi E. Pruiksma, David A. Moore, Lily Jankowiak, Hsing-Fang Hsieh, Philip Cheng, Sattvik Basarkod, Christopher L. Drake

**Affiliations:** 1 Sleep Disorders & Research Center, Henry Ford Health, Detroit, MI 48202, USAcdrake1@hfhs.org (C.L.D.); 2 Department of Psychiatry, Michigan State University College of Human Medicine, Grand Rapids, MI 49503, USA; 3 Department of Surgery, Division of Acute Care Surgery, Henry Ford Hospital, Detroit, MI 48202, USA; 4 Department of Psychiatry and Behavioral Neurosciences, Wayne State University School of Medicine, Detroit, MI 48202, USA; 5 Department of Psychiatry and Behavioral Sciences, The University of Texas at San Antonio, San Antonio, TX 78249, USA; 6 Department of Psychiatry and Behavioral Health, Division of Consultation Liaison Psychiatry, Henry Ford Hospital, Detroit, MI 48202, USA; 7 School of Public Health, University of Michigan, Ann Arbor, MI 48109, USA

**Keywords:** trauma-induced insomnia, trauma-related sleep disturbances, sleep health, neighborhood stress, urban trauma, hypervigilance, nocturnal arousal, longitudinal

## Abstract

Research suggests fear of sleep (FoS) may be an important consequence of trauma that increases risk for posttraumatic stress disorder (PTSD), especially among patients experiencing ongoing threat after their trauma has ended. Community violence exposure may reinforce perceptions of threat, compounding the pathogenic effect of FoS after trauma. The current study investigated whether FoS increases within the acute aftermath of trauma, and if such increases in FoS predict future PTSD severity. Further, we tested whether community violence exposure moderates the prospective relationship between FoS and PTSD. We recruited patients from an urban Level I trauma center (*N* = 88; *M*_age_ = 39.53 ± SD 14.31, 67.0% male, 67.0% Black). Patients reported FoS within one week of trauma (T1) and again one month later (T2), and PTSD symptoms two months later (T3). We operationalized community violence exposure as the frequency of hearing gunshots in the 90 days prior to trauma. FoS significantly increased from T1 (*M* = 8.80) to T2 (*M* = 11.98), *p* = 0.015, *g* = 0.28. Change in FoS significantly predicted PTSD symptoms at T3, and this effect was most pronounced among patients who frequently heard gunshots in their community (β = 0.61, *SE* = 0.35, *p* = 0.005). Exploratory analyses in a subsample of patients revealed preliminary associations between skin conductance reactivity and sleep fears at T1, tentatively suggesting heightened sympathetic activation as a corollary of fear of sleep. This study provides novel evidence that FoS increases in response to acute trauma exposure and, in turn, predicts future PTSD severity. Moreover, patients exposed to community violence may be especially vulnerable to these effects, perhaps due in part to continued threats to safety. Acute trauma patients who develop sleep fears may be vulnerable to PTSD, particularly those returning to neighborhoods marked by high levels of community violence.

## 1. Introduction

Acute trauma exposure can imbue life’s most ordinary activities with an ambient sense of danger. In the days and weeks after a traumatic event, routine tasks such as driving or walking to the store may suddenly feel life-threatening. For some, trauma exposure may even affect the act of going to sleep, turning it into a stressful and frightening experience. By its nature, falling asleep requires a relinquishing of control and vigilance. This process of surrendering to sleep creates an inherently vulnerable state that might be highly distressing in the aftermath of trauma, thus giving rise to a *fear of sleep*.

Fear of sleep reflects a fear of being vulnerable and unsafe during sleep (Werner et al., 2021). Individuals with a fear of sleep maintain hypervigilance at bedtime that increases their perceived safety, such as repeatedly checking the locks on the windows and doors at night and sleeping with the lights on (Pruiksma et al., 2014). This hypervigilance is incompatible with sleep, however, and sustains nocturnal arousal that further impairs sleep (Werner et al., 2020, 2021; Zayfert & DeViva, 2004). For instance, fear of sleep is associated with shorter sleep duration (Hall Brown & Mellman, 2014; Kanady et al., 2018), reduced sleep efficiency (Short et al., 2018), worse sleep quality (Correia, 2024; Short et al., 2018), and disruptive nocturnal behaviors (e.g., trouble sleeping due to general nervousness) (Kanady et al., 2018). Consequently, a fear of sleep that emerges *immediately post-trauma* may create a vulnerability to posttraumatic sequelae commonly downstream of sleep disturbances, namely posttraumatic stress disorder (PTSD) (Pigeon & DeViva, 2021).

Indeed, healthy sleep after trauma appears critical to halt the progression to PTSD among acute trauma patients (Koren et al., 2002; Mellman et al., 2002, 2007; Mellman & Hipolito, 2006; Neylan et al., 2021; Reffi et al., 2025b). However, the act of going to sleep shortly after a trauma may evoke intense fear and anxiety because doing so requires a loss of vigilance that may be adaptive after a life-threatening event to minimize vulnerability to future threats (Woodward, 1995). Over time, this fear of sleep could fuel a vicious cycle of hypervigilance and nocturnal arousal, eroding sleep in the aftermath of trauma and engendering risk for PTSD. In fact, fear of sleep has been associated with PTSD across chronically traumatized populations, such as first responders (Reffi et al., 2023) and veterans (Hull et al., 2016). Although fear of sleep is theorized to be a reaction to trauma exposure that may increase vulnerability to PTSD (Pigeon & DeViva, 2021), to our knowledge, no research has tested whether fear of sleep increases immediately after trauma, and if such increases confer a future risk for PTSD.

A fear of sleep may be especially pathogenic among patients exposed to the potential for ongoing threat after trauma (Zayfert & DeViva, 2004). For instance, residents of communities with high rates of violence perceive greater threats to their own safety (Semenza & Kravitz-Wirtz, 2024). As such, trauma survivors returning to neighborhoods marked by community violence may be exceptionally hypervigilant. Indeed, trauma patients who frequently hear gunshots in their neighborhood may feel keenly vulnerable and unsafe in their home (Hall Brown & Mellman, 2014; Miller et al., 2023; Poindexter et al., 2023). This sense of ongoing threat may reinforce hypervigilance, especially at nighttime (Mellman et al., 2018), when gunshots are more likely to occur (Robbins et al., 2024). Consequently, those living in violent communities may have an overactive arousal system primed for nocturnal hypervigilance, leading to poorer sleep compared to those who live in safer communities (Mellman et al., 2018). A fear of sleep that emerges within a threatening context may therefore be more damaging for these patients, fueling nocturnal hypervigilance that further degrades sleep after trauma, and thus increasing the likelihood of developing PTSD.

Taken together, the literature points to fear of sleep as an important consequence of trauma that may impact recovery, especially for those who perceive ongoing threats to their safety (Miller et al., 2023). However, several gaps in the literature remain. First, despite being posited as a reaction to trauma, no prospective data exists showing that fear of sleep increases following trauma. Second, although fear of sleep as a reaction to trauma has been suggested to increase PTSD risk, no studies have examined whether fear of sleep in the acute aftermath of trauma prospectively predicts future PTSD. Third, no studies have examined how exposure to community violence may affect the relationship between fear of sleep and PTSD. These are critical gaps in research because fear of sleep is modifiable with treatment (Kanady et al., 2018; Pigeon et al., 2025), suggesting it could be targeted early in acute settings to potentially prevent PTSD. Moreover, identifying populations most vulnerable to PTSD after increased fear of sleep will highlight those most in need of early intervention efforts (i.e., those living in high-violence communities).

The purpose of the current prospective study, therefore, was to investigate changes in fear of sleep in the acute aftermath of trauma as a predictor of PTSD and its potential interaction with community violence exposure. We recruited patients from an urban Level I trauma center in Detroit, MI, USA, immediately within one week post-trauma exposure, and followed them at one and two months post-trauma. This setting is significant because exposure to community violence is pervasive in low-income urban environments and might be a social determinant of sleep health that disrupts trauma recovery in this population (Billings et al., 2021; Robbins et al., 2024; Semenza & Kravitz-Wirtz, 2024). Our hypotheses were as follows: (1) fear of sleep would increase from one week to one month post-trauma, (2) increases in fear of sleep would predict future PTSD symptoms two months post-trauma, and (3) the association between increases in fear of sleep and future PTSD would be strongest among patients with high exposure to community violence. Finally, we preliminarily tested physiological arousal as a corollary of fear of sleep by measuring skin conductance response on a subsample of patients. We carried out this exploratory analysis because nocturnal arousal is one putative mechanism by which fear of sleep might confer PTSD risk (Gupta & Sheridan, 2018; Werner et al., 2020); thus, identifying sympathetic activation as a potential biomarker of fear of sleep may help inform future research on treatment and prevention strategies.

## 2. Method

### 2.1. Participants and Procedure

We recruited participants from the surgical general practice units, intensive care units, and emergency department within the Level I Trauma Center at Henry Ford Hospital in midtown Detroit, MI, USA (Reffi et al., 2024a). Patients were recruited following trauma exposure between April 2022 and January 2023. Inclusion criteria were as follows: ≥18 years old and fluent in English. Regarding exclusion, patients were not approached if they had a traumatic brain injury, were intubated or sedated, actively recovering from anesthesia, or experiencing delirium. This study was approved by Henry Ford Health’s (HFH) Institutional Review Board.

We recruited trauma-exposed patients from the hospital (Time 1 [T1]; *M*_days after trauma_ = 5.23 ± *SD* 4.42) and prospectively followed them to one month (Time 2 [T2]; *M*_days after trauma_ = 24.28 ± *SD* 7.53) and two months (Time 3 [T3]; *M*_days after trauma_ = 47.81 ± *SD* 17.94) post-trauma follow-ups. Study personnel recruited and obtained informed consent from eligible patients within one week of trauma exposure. Patients were informed during the initial contact and consent process of the potential to receive monetary compensation for their participation. Eighty-eight interested patients completed the T1 survey in person in the hospital using Qualtrics (Qualtrics, Provo, UT, USA). We measured skin conductance on a subsample of patients (*n* = 7) during part of the T1 survey while they were still in the hospital (described in detail below). Participants completed follow-up Qualtrics surveys via email at T2 (*n* = 61) and T3 (*n* = 59). After T3, participants were compensated monetarily for each survey they completed and also received a list of regional mental health resources.

### 2.2. Measures

#### 2.2.1. Fear of Sleep

Participants completed the fear of sleep inventory short form (FoSI-SF) to indicate their fear of sleep at one week post-trauma (T1) and one month post-trauma (T2) (Pruiksma et al., 2014). The FoSI-SF is a 13-item questionnaire that assesses fears in relation to sleep (e.g., “I was fearful of letting my guard down while sleeping”) and safety behaviors to cope with such fear (e.g., “I slept with a light on to feel safer”). Participants indicated how often they experienced sleep fears or engaged in associated safety behaviors over the past month at both T1 and T2 using a 5-point scale ranging from 0 (not at all) to 4 (nearly every night), for a possible total score range of 0–52, with greater sum scores indicating more severe fear of sleep. We computed a fear of sleep change score to use as our predictor variable by subtracting the FoSI-SF score at the one-month assessment from the score at the one-week assessment (T2 FoSI-SF–T1 FoSI-SF). This method of calculating the difference between scores is common for assessing change between two time points (Singer & Willett, 2003). The internal consistency for the FoSI-SF in this study was good at T1 (α = 0.88) and excellent at T2 (α = 0.94). Scores on the FoSI-SF were moderately correlated with each other from T1-T2 (*r* = 0.52, *p* < 0.001).

For descriptive purposes, participants also completed four supplemental items at T1 to report their history of potentially traumatic experiences that occurred in a sleep context (e.g., “Dangerous, frightening, or very unpleasant things have happened to me while I was in bed.”) (see Supplemental Table S1). These types of traumatic events are theorized to engender a fear of sleep; thus, we conducted exploratory analyses to compare fear of sleep scores between patients who endorsed a history of experiencing traumatic events in a sleep context versus those who did not (see Supplemental Table S2).

#### 2.2.2. Skin Conductance Response to Fear of Sleep

We measured skin conductance response (SCR) among a subsample of patients (*n* = 7) as an index of sympathetic activation while they simultaneously completed the FoSI-SF in the hospital at T1. We measured skin conductance using eSense (Mindfield Biosystems, Inc., Berlin, Germany), an app that records skin conductance level (SCL) via an iPad tablet. Prior to the FoSI-SF, we applied isotonic electrode paste and electrodes to the patients’ non-dominant index and middle fingers. We then connected the electrodes to an iPad via a headphone jack cable. We collected skin conductance data using the eSense device at a sampling rate of 5Hz. We recorded a 2-min baseline to establish the patients’ resting SCL while they sat quietly and remained calm. Following the baseline, the patients were administered the FoSI-SF via interview while skin conductance was continuously recorded. This method of SCL data collection has been externally validated against gold standard psychophysiological recording equipment in prior research (Hinrichs et al., 2017). We quantified the patients’ SCR by calculating the maximum SCL during the interview and subtracting from it the last 30 s of the baseline SCL (SCL_max_ − SCL_baseline_), in line with previous studies (Hinrichs et al., 2017, 2019).

#### 2.2.3. Community Violence Exposure

Participants reported their exposure to community violence in the 90 days prior to trauma using the single item on community violence from the SaFETy questionnaire at T1 (Goldstick et al., 2017). This questionnaire operationalizes community violence exposure as frequency of hearing gunshots using the following prompt: “In this survey, when we say guns, we mean guns that are in working order and capable of being fired. This includes pistols, revolvers, shotguns, and rifles, but does not include air guns, BB guns, starter pistols, or paintball guns. In the past three months, including today, how often have you heard guns being shot?” This item was originally included in the SaFETy questionnaire as an assessment of community violence exposure to increase the likelihood of honest responding in clinical settings, relative to other items that might promote underreporting (e.g., asking patients about how often they witnessed someone being shot) (Goldstick et al., 2017).

Participants indicated how frequently they heard gunshots in the 90 days prior to their trauma using the following response options: never, once or twice, a few times, and many times. Consistent with the SaFETy scoring (Goldstick et al., 2017), we computed a dichotomous variable where 0 = no or low exposure to community violence (heard gunshots never, once or twice, or a few times) and 1 = high exposure to community violence (heard gunshots many times) to be used as our moderator variable. This recommended cut-off has been validated in urban trauma populations for its ability to identify those at highest risk for community violence exposure (Goldstick et al., 2017).

#### 2.2.4. PTSD Symptoms

Participants completed the Posttraumatic Stress Disorder Checklist for DSM-5 (PCL-5) at T1 and T3. The PCL-5 is a 20-item questionnaire that assesses PTSD symptoms in relation to a traumatic event using a 5-point scale ranging from 0 (not at all) to 4 (extremely) (Weathers et al., 2013b). Participants completed the PCL-5 at T1 to indicate their PTSD symptoms over the past month in relation to their worst lifetime trauma as indicated on the Life Events Checklist for DSM-5 (Weathers et al., 2013a). We computed a dichotomous variable using a cutoff score of ≥33 to indicate PTSD status at T1 to be used as a covariate (Bovin et al., 2016). Participants then completed the PCL-5 two months post-trauma (T3) to indicate their PTSD symptoms over the past month in relation to the trauma that precipitated their hospital visit. We computed a sum score to be used as our outcome, with greater scores indicating more severe PTSD symptoms two months post-trauma (possible range of 0–80).

## 3. Data Analysis Plan

To test our first hypothesis, we ran a paired samples t-test to assess mean differences in fear of sleep scores from the initial assessment, one week post-trauma (T1), to the first follow-up assessment one month post-trauma (T2).

To test our second hypothesis, we performed a linear regression analysis testing change in fear of sleep (T2 − T1) as a prospective predictor of PTSD symptoms two months post-trauma (T3).

To test our third hypothesis, we performed a moderation analysis testing the interaction between exposure to community violence (T1) and change in fear of sleep (frequently hearing gunshots × T2 − T1 fear of sleep) on future PTSD severity (T3).

Finally, we assessed exploratory cross-sectional correlations between SCR and the FoSI-SF and its individual items (all collected at T1).

In our regression models, we adjusted for T1 PTSD status and days elapsed since acute trauma exposure and assessed demographic characteristics as potential covariates by examining their bivariate associations with our outcome. We retained demographic variables as covariates if they were significantly correlated with our outcome at *p* < 0.05 and significantly improved model fit (significant ∆F) (Cohen et al., 2013). We report β’s to denote standardized beta coefficients, Hedge’s *g* to indicate effect sizes, with *g* values of 0.20 = small, 0.50 = medium, 0.80 = large, and 1.30 = very large (Sullivan & Feinn, 2012), and Kendall’s Tau-b (*τb*) to assess correlations between SCR and the FoSI-SF, a non-parametric test more suitable for small sample sizes than Pearson’s *r* (Field, 2018).

## 4. Results

### 4.1. Demographics

Most participants reported being male (*n* = 59, 67.0%, *M*_age_ = 39.53), Black or African American (*n* = 59, 67.0%), and nearly half indicated earning an annual household income of $20,000 or less (*n* = 42, 47.7%). See Table 1 for complete demographic data on the full sample^1^.

### 4.2. Trauma Types That Precipitated Hospital Visit

Patients most often presented to the hospital after a motor vehicle collision (*n* = 37, 42.0%), followed by an assault with a weapon (*n* = 27, 30.7%), fall (*n* = 8, 9.1%), physical assault (*n* = 6, 6.8%), self-injury (*n* = 2, 2.3%), and sexual assault (*n* = 1, 1.1%).

### 4.3. Rates of Community Violence Exposure

Three-quarters of our sample reported any exposure to community violence in the 90 days prior to their most recent trauma (*n* = 66, 75% heard gunshots more than “never”), and over one-third reported high exposure to community violence (*n* = 35, 39.8% heard gunshots “many times”)^2^.

### 4.4. Changes in Fear of Sleep After Trauma

**Figure 1:**
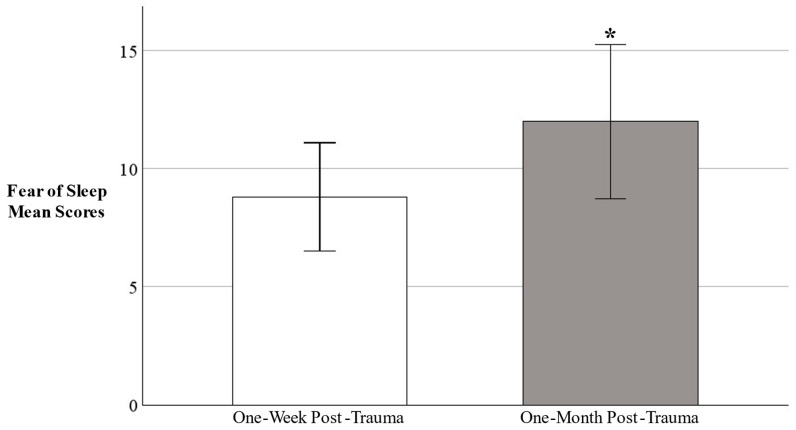
Fear of Sleep Increases Immediately Post-Trauma. Note: Patients completed the Fear of Sleep Index Short Form at approximately one-week after trauma (*M*_days_ = 5.23) and one-month after trauma (*M*_days_ = 24.28). * *p* < 0.05. Error bars represent 95% confidence intervals.

First, we examined whether fear of sleep scores significantly changed within the acute aftermath of trauma. A paired samples *t*-test indicated fear of sleep significantly increased from the one-week post-trauma assessment (*M* = 8.80 ± *SD* 8.93) to one-month post-trauma assessment (*M* = 11.98 ± *SD* 12.74), *p* = 0.015, *g* = 0.28 (see Figure 1).

### 4.5. Fear of Sleep as a Predictor of Future PTSD

Next, we tested the change in fear of sleep post-trauma as a prospective predictor of PTSD symptoms two months after trauma. Sex was the only significant demographic covariate and thus was retained in subsequent regression analyses^3^. Change in fear of sleep was a significant predictor of future PTSD symptoms (β = 0.26, *SE* = 0.18, *p* = 0.043), independent of T1 PTSD status, sex, and days since trauma. The main effect of fear of sleep explained 43% of variance in PTSD symptoms (adjusted *R*^2^ = 0.431). The full model summary is displayed in Table 2.

### 4.6. Community Violence Exposure as a Moderator of Fear of Sleep on Future PTSD

We then tested whether high exposure to community violence in the 90 days prior to trauma moderated the association between changes in fear of sleep and future PTSD symptoms (frequently hearing gunshots × T2 − T1 fear of sleep). We found a significant interaction effect, such that high exposure to hearing gunshots strengthened the effect of changes in fear of sleep on future PTSD (β = 0.40, *SE* = 0.38, *p* = 0.003), independent of T1 PTSD status, sex, and days since trauma.

Specifically, for patients reporting hearing gunshots many times prior to trauma, increases in fear of sleep prospectively predicted more severe PTSD (β = 0.61, *SE* = 0.35, *p* = 0.005). However, for patients hearing gunshots a few times or never prior to trauma, the relationship between an increase in fear of sleep post-trauma and future PTSD was nonsignificant (β = 0.14, *SE* = 0.17, *p* = 0.309). The full model, including all predictors and the interaction term, explained 53% of the variance in PTSD symptoms (adjusted *R*^2^ = 0.533). The full model summary is displayed in Table 3.

### 4.7. Preliminary Evidence of Sympathetic Arousal as a Psychophysiological Correlate of Fear of Sleep

**Figure 2:**
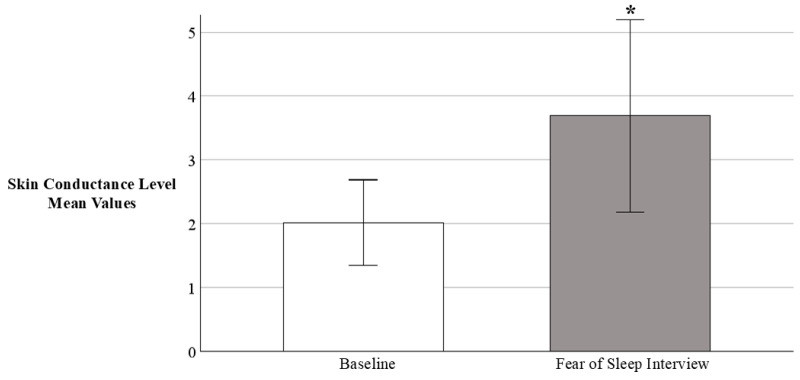
Skin Conductance Response to the Fear of Sleep Inventory. Note: Skin conductance levels (SCL) were measured on a subsample of patients (*n* = 7) while they completed the Fear of Sleep Inventory Short Form (FoSI-SF) via interview during their hospital visit approximately one-week after trauma (*M* days = 5.23). Baseline = average SCL during last 30 s of baseline, Fear of Sleep Interview = peak SCL during FoSI-SF interview. SCL measured in microSiemens. * *p* < 0.05. Error bars represent 95% confidence intervals.

We measured skin conductance levels (SCLs) on a subsample of patients (*n* = 7)^4^ while asking them about their fear of sleep using the FoSI-SF during their hospital visit (T1). Patients demonstrated a significant skin conductance response (SCR) to the FoSI-SF interview, as evidenced by an increase in SCL_baseline_ (*M* = 2.02 ± *SD* 0.73) to SCL_max_ (*M* = 3.69 ± *SD* 1.63), *p* = 0.025, *g* = 0.80 (see Figure 2).

We found a trend-level association between greater SCR and higher FoSI-SF total scores (*τb* = 0.55, *p* = 0.091). Greater SCR was significantly correlated with the following fear-of-sleep-related items: “*I was fearful of letting my guard down while sleeping*” (*τb* = 0.72, *p* = 0.029) and “*I stayed up late to avoid sleeping*” (*τb* = 0.79, *p* = 0.017). We also observed trend-level correlations between SCR and the following FoSI-SF items: “*I felt that it was dangerous to fall asleep*” (*τb* = 0.62, *p* = 0.065), “*I woke up in the night and I was terrified of returning to sleep*” (*τb* = 0.58, *p* = 0.081), and “*I was aware of being especially vulnerable when I’m asleep*” (*τb* = 0.55, *p* = 0.091).

## 5. Discussion

Research has increasingly pointed to fear of sleep as a clinically meaningful consequence of traumatic exposure that is associated with PTSD (Werner et al., 2021). However, no research has tested whether fear of sleep increases immediately post-trauma, and if such increases confer a future risk for PTSD. The current study recruited acute trauma patients from an urban Level I trauma center and found novel evidence that fear of sleep significantly worsened from one week to one month post-trauma, and this increase in sleep fears predicted future PTSD symptoms two months post-trauma. Notably, increases in fear of sleep after trauma predicted future PTSD symptoms most strongly for patients with high exposure to community violence in the 90 days prior to trauma. Changes in fear of sleep after trauma might predict subsequent PTSD severity in part because of nocturnal hypervigilance and arousal, exacerbated by community violence exposure.

### 5.1. Fear of Sleep Increases from One Week to One Month Post-Trauma

Fear of sleep reflects a fear of being vulnerable and unsafe during sleep. It is unsurprising, then, that our patients experienced an increase in fear of sleep in the initial weeks after trauma, a time during which they likely still felt deprived of their sense of safety. Exposure to trauma can change a person’s beliefs about the world, such as inflating the perceived level of threat present across situations (Janoff-Bulman, 1989). For example, survivors of motor vehicle collisions may overestimate the likelihood of another crash each time they enter a vehicle. Our findings suggest that these trauma-related changes in perceived vulnerability and insecurity may generalize to the act of going to sleep, subverting this otherwise benign experience into something precarious and foreboding (e.g., “I felt that it was dangerous to fall asleep”) (Woodward, 1995).

### 5.2. Increases in Fear of Sleep Post-Trauma Predict Future PTSD Symptoms

The observed increase in fear of sleep from one week post-trauma to one month post-trauma predicted more severe PTSD symptoms two months post-trauma. This finding may be explained in part by the detrimental impact that fear of sleep has on sleep, ranging from deliberate efforts to avoid sleep to difficulty inhibiting arousal at bedtime (Werner et al., 2020). Indeed, our preliminary associations between SCR and sleep fears tentatively suggest heightened sympathetic activation may underlie a fear of sleep, consistent with evidence that somatic pre-sleep arousal is an important corollary of fear of sleep (Reffi et al., 2024b; Werner et al., 2020). When occurring immediately after trauma, these sleep fears and the resulting nocturnal arousal may lead to inadequate and poor-quality sleep that undermines the ability to cope with or emotionally process the traumatic event (Colvonen et al., 2019; Walker & van der Helm, 2009). This novel finding connects a broad literature documenting sleep disturbance after trauma as a predictor of posttraumatic sequelae with emerging evidence that fear of sleep is associated with PTSD and a host of other trauma-related outcomes (Mellman & Hipolito, 2006; Reffi et al., 2023, 2025a, 2025b; Werner et al., 2021).

### 5.3. Community Violence Exposure Amplifies the Impact of Fear of Sleep on PTSD Symptoms

We also found that high exposure to community violence in the 90 days prior to trauma compounded the impact of fear of sleep after trauma on PTSD. An increase in fear of sleep immediately after trauma might engender nocturnal hypervigilance that prevents the offset of the stress response in the evening, impeding the transition to sleep that in turn enhances risk for later PTSD. This effect may be even more pronounced among trauma patients who return to a home where they perceive themselves to be under continued threat.

For instance, patients who reenter environments with ongoing exposure to violence immediately post-trauma may feel a pervasive sense of danger in their own home. Compared to patients reporting a history of no to low exposure to hearing gunshots, those exposed to the frequent sound of gunshots in their community may have a highly sensitized, nocturnal stress response that makes it substantially more difficult to downregulate sleep fears and the associated nocturnal hypervigilance (Miller et al., 2023). A fear of sleep that emerges within this context might therefore trigger a robust stress response that persists overnight, even during sleep (Mellman et al., 2018), significantly deteriorating sleep in the immediate wake of trauma and compounding vulnerability to subsequent PTSD. Exposure to frequently hearing gunshots may also exacerbate the impact of fear of sleep on PTSD because gunshots are more often heard at nighttime (Robbins et al., 2024), potentially reinforcing sleep fears (e.g., “I was fearful of letting my guard down while sleeping”) (Miller et al., 2023; Poindexter et al., 2023) and making them more entrenched rather than allowing them to extinguish naturally.

### 5.4. Clinical Implications

Our findings shed light on fear of sleep after trauma as a potential early predictor of PTSD, and the compounding effect of community violence exposure. As such, treatments that effectively target fear of sleep within the acute aftermath of trauma may promote recovery. There is evidence that cognitive behavioral therapy for insomnia (CBT-I) effectively reduces fear of sleep (Kanady et al., 2018), which in turn mediates improvement in PTSD (Pigeon et al., 2025). Moreover, CBT-I may be appropriate for patients returning to threatening environments that could reinforce sleep fears. For instance, at least one study has shown that CBT-I effectively improves sleep and PTSD among patients with potential for ongoing safety concerns (intimate partner violence) (Pigeon et al., 2022). The question of the most appropriate intervention(s) for addressing fear of sleep as a preventive strategy among acute trauma patients exposed to community violence is an area ripe for future investigation.

### 5.5. Limitations and Strengths

Our study has several notable limitations. Our sample size was relatively small and showed attrition over time. Although attrition is commonly observed in acutely traumatized samples, it is important to replicate these findings using larger datasets. Additionally, it is possible that we captured early fear of sleep reactions that would have worsened over repeated assessments. For instance, we observed a small increase in fear of sleep from one week post-trauma to one month post-trauma (*g* = 0.28), perhaps because fear of sleep takes more time to develop. Conversely, we may have also captured individuals whose fear of sleep would have remitted over a longer timeframe. Thus, it will be important to assess changes in fear of sleep over longer periods of time post-trauma.

Regarding measurement, our assessment of community violence exposure was limited to a single item on frequency of hearing gunshots and may have benefited from assessing more dimensions of neighborhood disorder (e.g., illicit substance activity, urban blight) (Miller et al., 2023; Poindexter et al., 2023). Future researchers are encouraged to build on our findings using more comprehensive measures of community violence, such as the Neighborhood Stress Index (Ewart & Suchday, 2002) or the Survey of Exposure to Community Violence (Richters & Saltzman, 1990). Our findings on SCR and fear of sleep are from a small subsample and should be considered preliminary. Still, the observed associations tentatively suggest heightened sympathetic activation may underlie a fear of sleep, which builds on prior self-report data pointing to somatic pre-sleep arousal as a distinguishing feature of fear of sleep (Werner et al., 2020). It is also possible that the increased SCR we observed from the 2-min baseline to the fear of sleep interview was partly due to participants interacting with study personnel and perhaps experiencing general task-related tension related to answering questions. That said, this method of SCL data collection has been externally validated against gold standard psychophysiological recording equipment in prior research (Hinrichs et al., 2017).

Our study also has strengths. This is the first study to evaluate fear of sleep within the acute aftermath of trauma, including how fear of sleep changes immediately post-trauma and predicts subsequent PTSD severity. Research on fear of sleep has received increasing attention recently, with researchers suggesting fear of sleep after trauma exposure may be important in the development of PTSD (Pigeon & DeViva, 2021). This study provides novel support for this pathway, suggesting that addressing sleep fears early in the wake of trauma may help disrupt the progression to PTSD. Additionally, our study underscores the role of community violence exposure in compounding the effect of fear of sleep on PTSD. This is an important contribution as it adds to our understanding of how sleep health is impacted by multiple factors that span the individual, family, neighborhood, and broader socio-cultural context (Billings et al., 2021).

## 6. Conclusions

The current longitudinal study found increases in fear of sleep from one week post-trauma to one month post-trauma prospectively predicted more severe PTSD symptoms two months post-trauma. This relationship was strongest among patients with high exposure to community violence prior to trauma. Fear of sleep may foster a state of chronic stress activation around bedtime, especially among patients exposed to high levels of community violence, sustaining nocturnal hypervigilance in the acute aftermath of trauma that promotes PTSD. Presenting with sleep fears following acute trauma may serve as a marker for PTSD risk, especially among patients from disadvantaged communities. Behavioral sleep interventions delivered early after trauma may help survivors regain a healthy relationship with sleep that, in turn, could activate downstream mental health benefits.

## Figures and Tables

**Table 1:** Demographics (*N* = 88).

Variables	*n* (%)
Age (*M* ± *SD*)	39.5 (14.3)
Sex (male)	59 (67.0%)
Race ^1^	
Black or African American	59 (67.0%)
White	29 (33.0%)
Other	4 (4.4%)
Hispanic/Latino(a)	3 (3.4%)
Highest level of education	
Some college credit, no degree	27 (30.7%)
High school diploma/GED	32 (36.4%)
Some high school, no diploma	13 (14.8%)
Other	16 (18.1%)
Working full-time	36 (40.9%)
Annual household income	
≤20 k	42 (47.7%)
≤50 k	22 (25.0%)
≤75 k	16 (18.2%)
≤100 k	1 (1.1%)
>100 k	7 (8.0%)
Relationship status	
Single	50 (56.8%)
Never married, in relationship	18 (20.5%)
Married	8 (9.1%)
Separated or divorced	12 (13.6%)

^1^ Participants could select multiple responses.

**Table 2:** Linear regression analysis modeling change in fear of sleep after trauma as a prospective predictor of future PTSD symptoms.

	*b* (*SE*)	β	*p*	*R* ^2^	*Adj R* ^2^
Model				0.49	0.43
Sex	−11.56 (4.41)	−0.33	0.013		
Days since trauma	0.48 (0.15)	0.41	0.002		
T1 PTSD	10.83 (5.31)	0.25	0.049		
T2-T1 FoS	0.38 (0.18)	0.26	0.043		

Note. Summary of final model after including each variable as a prospective predictor of future PTSD symptoms (PTSD Checklist for DSM-5 (PCL-5) sum score completed approximately two months post-trauma). Sex = female (0) and male (1); T1 PTSD = PCL-5 score ≥ 33 completed approximately one week post-trauma; T2-T1 FoS = change in fear of sleep from approximately one week post-trauma (T1) to approximately one month post-trauma (T2); *b* = unstandardized regression coefficient; *SE* = standard error; β = standardized regression coefficient; *p* = significance value; *R*^2^ = amount of variance in PTSD symptoms explained by the model (adjusted *R*^2^ corrects for the number of predictors in the model).

**Table 3:** Moderation analysis modeling the interaction between community violence exposure and change in fear of sleep after trauma on future PTSD symptoms.

	*b* (*SE*)	β	*p*	*R* ^2^	*Adj R* ^2^
Model				0.60	0.53
Sex	−11.10 (4.46)	−0.32	0.018		
Days since trauma	0.46 (0.14)	0.40	0.002		
T1 PTSD	9.15 (4.89)	0.21	0.070		
T2-T1 FoS	0.10 (0.19)	0.07	0.613		
T1 CVE	−2.55 (4.34)	−0.07	0.561		
T1 CVE × T2 − T1 FoS	1.19 (0.38)	0.40	0.003		

Note. Summary of final model after including each variable as a prospective predictor of future PTSD symptoms (PTSD Checklist for DSM-5 (PCL-5) sum score completed approximately two months post-trauma). Sex = female (0) and male (1); T1 PTSD = PCL-5 score ≥ 33 completed approximately one week post-trauma; T2-T1 FoS = change in fear of sleep from approximately one week post-trauma (T1) to approximately one month post-trauma (T2); T1 CVE = community violence exposure in the 90 days prior to trauma operationalized as frequency of hearing gunshots (“In the past three months, including today, how often have you heard guns being shot?”) dichotomized into no or low exposure (0) and high exposure (1); T1 CVE × T2 − T1 FoS = interaction between community violence exposure and change in fear of sleep after trauma. *b* = unstandardized regression coefficient; *SE* = standard error; β = standardized regression coefficient; *p* = significance value; *R*^2^ = amount of variance in PTSD symptoms explained by the model (adjusted *R*^2^ corrects for the number of predictors in the model).

## Data Availability

The raw data supporting the conclusions of this article will be made available by the authors upon request.

## Notes

1 Compared to the U.S. Census data for Detroit, MI, our sample slightly overrepresented male patients and slightly underrepresented Hispanic patients, but was otherwise comparable to the population’s demographic estimates (*U.S. Census Bureau QuickFacts*, 2025).

2 Male patients reported the highest rates of community violence exposure (47.5%) relative to female patients (24.1%, Relative Risk = 1.97, χ^2^ = 4.41, *p* = 0.036). Correlations and chi-square analyses revealed that high exposure to community violence was not significantly associated with other patient demographics (age, race, income) nor the trauma types that precipitated hospital visits (interpersonal [e.g., assault] vs non-interpersonal [e.g., motor vehicle collisions]), *p*s > 0.05.

3 Female sex was significantly associated with more severe PTSD symptoms two months post-trauma at the bivariate level (*r* = −0.35, *p* = 0.016) and uniquely accounted for 17% of its variance in the regression model (adjusted *R*^2^ = 0.172, *p* = 0.004).

4 We report skin conductance data from only a subsample of our participants because we added this data collection method toward the end of our study, when recruitment was winding down. Out of an initial subsample of 23 patients, we experienced technical difficulties with our mobile skin conductance device with 9 patients and observed significant abnormalities in the data from 7 patients (e.g., due to someone entering the hospital room during collection), resulting in a final subsample of 7 usable data points.

## Supplementary Materials

The following supporting information can be downloaded at: https://www.mdpi.com/article/10.3390/bs16030443/s1, Table S1: Potentially traumatic experiences occurring in a sleep context. Table S2: Independent samples *t*-tests comparing fear of sleep after trauma by a history of potentially traumatic experiences occurring in a sleep context.
