# Supplementary material for: Fear of Sleep in the Acute Aftermath of Trauma Predicts Future Posttraumatic Stress Disorder: The Moderating Role of Community Violence Exposure
Source: Behav Sci (Basel). 2026 Mar 18;16(3):443. doi: 10.3390/bs16030443 (PMC13023504; doi:10.3390/bs16030443)
Supplement: Supplementary file 1 [file behavsci-16-00443-s001.zip › Supplemental Table S1_FoS experiences.pdf]

Supplemental Table S1 Potentially traumatic experiences occurring in a sleep context

Experience	*N* (%)	*M* age (*SD*)
Dangerous, frightening, or very unpleasant things have happened to me while I was in bed	27 (30.7%)	16.4 (11.7)
Dangerous, frightening, or very unpleasant things have happened to me while I was sleeping	31 (35.2%)	17.5 (11.4)
Dangerous, frightening, or very unpleasant things have happened to me in the dark	25 (28.4%)	15.9 (9.3)
There was a time when I had to stay on guard at night or while I was in bed in order to protect myself or others	43 (48.9%)	19.9 (12.5)

Note. Participants reported these experiences (true/false) and the age at which they occurred within one week of acute trauma (during their hospital visit).
