# Supplementary material for: Fear of Sleep in the Acute Aftermath of Trauma Predicts Future Posttraumatic Stress Disorder: The Moderating Role of Community Violence Exposure
Source: Behav Sci (Basel). 2026 Mar 18;16(3):443. doi: 10.3390/bs16030443 (PMC13023504; doi:10.3390/bs16030443)
Supplement: Supplementary file 1 [file behavsci-16-00443-s001.zip › Supplemental Table S2_FoS t tests.pdf]

Supplemental Table S2 Independent samples t-tests comparing fear of sleep after trauma by a history of potentially traumatic experiences occurring in a sleep context

Experience		T1 FoSI-SF				T2 FoSI-SF				T2-T1 FoSI-SF			
		M (*SD*)	*t*	*p*	*g*	*M* (*SD*)	*t*	*p*	*g*	*M* (*SD*)	*t*	*p*	*g*
Dangerous, frightening, or very unpleasant things have happened to me while I was in bed	True	13.48 (10.33)	**-3.44**	**< .001**	**-0.79**	15.41 (12.18)	-1.60	.058	-0.42	3.27 (10.48)	-0.05	.481	-0.13
	False	6.38 (8.27)				10.05 (12.80)				3.13 (11.60)			
Dangerous, frightening, or very unpleasant things have happened to me while I was sleeping	True	14.19 (10.97)	**-4.00**[a]	**< .001**	**-1.01**	15.80 (11.95)	**-1.20**	**.025**	**-0.51**	2.04 (8.55)	0.67	.254	0.17
	False	5.49 (6.93)				9.33 (12.76)				3.97 (12.66)			
Dangerous, frightening, or very unpleasant things have happened to me in the dark	True	13.96 (12.10)	**-2.92**[a]	**.003**	**-0.84**	15.47 (12.45)	-1.45	.076	-0.40	1.42 (8.97)	0.83	.205	0.23
	False	6.41 (7.28)				10.40 (12.71)				3.98 (11.98)			
There was a time when I had to stay on guard at night or while I was in bed in order to protect myself or others	True	12.30 (10.22)	**-3.91**	**< .001**	**-0.83**	18.00 (13.94)	**-4.29**[a]	**< .001**	**-1.07**	5.16 (14.05)	-1.44[a]	.078	-0.36
	False	4.98 (7.15)				5.77 (7.51)				1.13 (6.55)			

Note. Significant differences are bolded ($p < .05$).

Participants reported their history of potentially traumatic experiences occurring in a sleep context (true/false) within one week of acute trauma (during their hospital visit). T1 FoSI-SF = fear of sleep sum score one-week post-trauma; T2 FoSI-SF = fear of sleep sum score one-month post trauma; T2-T1 FoSI-SF = change in fear of sleep sum score from one-week to one-month post trauma.

M = mean; *SD* = standard deviation; *t* = t-statistic; *p* = significance value; *g* = Hedges' *g* effect size: small = 0.2, medium = 0.5, large = 0.8.

[a]Equal variances not assumed.
